# Supplementary material for: Maternal histone methyltransferases antagonistically regulate autosomal random monoallelic expression (aRMAE) in C. elegans
Source: Nat Commun. 2025 Nov 28;16:11712. doi: 10.1038/s41467-025-66501-5 (PMC12753696; doi:10.1038/s41467-025-66501-5)
Supplement: Supplementary file 1 — Supplementary Information [file 41467_2025_66501_MOESM1_ESM.pdf]

Supplementary Information
for
Maternal histone methyltransferases antagonistically regulate autosomal random monoallelic expression (aRMAE) in *C. elegans*

Section 1. Supplemental Figures and Tables

Supplemental Figure 1. Detailed experimental workflow

Supplemental Figure 2. Quantification of MAE in *nrde-3(RNAi)*

Supplemental Figure 3. Antagonistic control of aRMAE by *met-2* and *set-25* in eef1A and intronless reporter strains.

Supplemental Figure 4. met-2 null suppressor screen

Supplemental Figure 5. Effects of *hpl-2* on *hsp-90* reporter allele expression in *met-2(null)* mutants

Supplemental Figure 6. Fecundity and lifespan on met-2 mutants

Supplemental Figure 7. Somatic RNAi initiation and inheritance are not affected by MAE regulators

Supplemental Figure 8. MAE is not heritable

Supplemental Figure 9. Sequencing of CRISPR mutants

Supplemental Figure 10. Longitudinal analysis

Supplemental Table 1. Strains

Supplemental Table 2. CRISPR details

Supplemental Table 3. Crosses

Section 2. Statistics

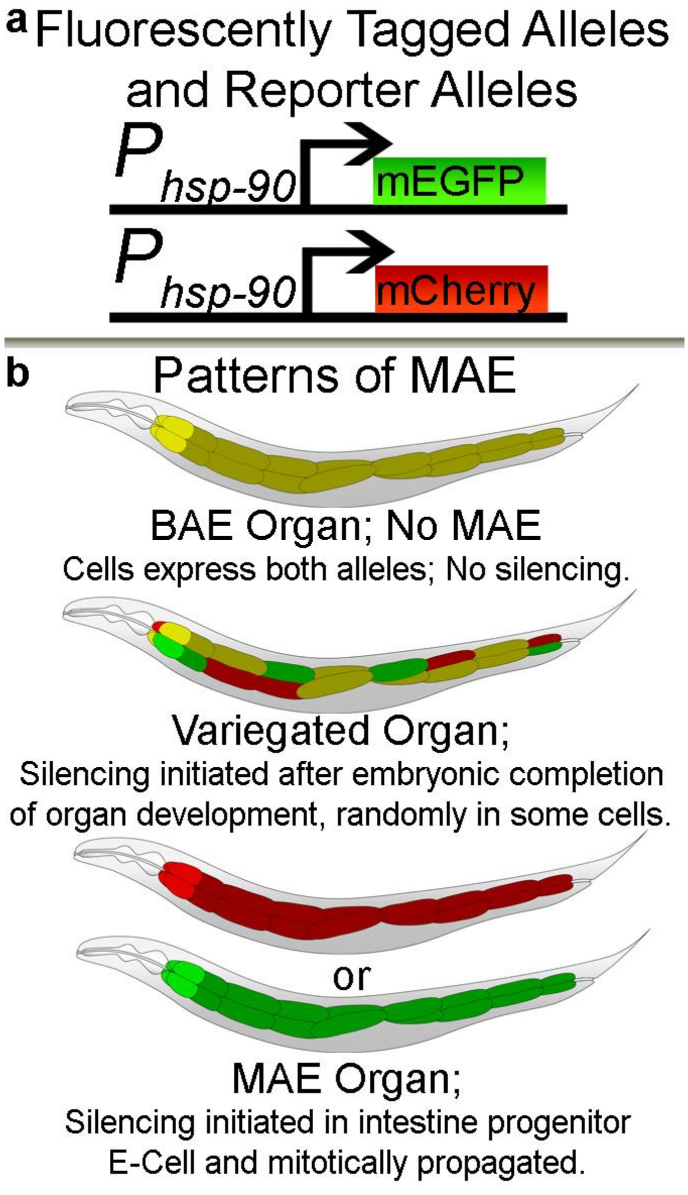

c

$$\text{Allele Bias} = \frac{(x-y)^2}{(2\langle x\rangle\langle y\rangle)}$$

Supplemental Figure 1. Animal Model for *In Vivo* Study of MAE, related to Figure 1. a) We tag native alleles or generated reporter alleles at identical loci. Schematic shows *hsp-90* promoter controlling expression of fluorescent reporter alleles. **b)** We quantify allele expression levels *in vivo* in the intestine cells of live *C. elegans* on a point scanning confocal microscope. Cartoons show biallelic expression and different ranges of monoallelic expression which exists on a spectrum from fully biallelic to fully monoallelic. **c)** We quantify the degree of allele expression bias using the intrinsic noise formula developed by Elowitz and Swain in 2002.

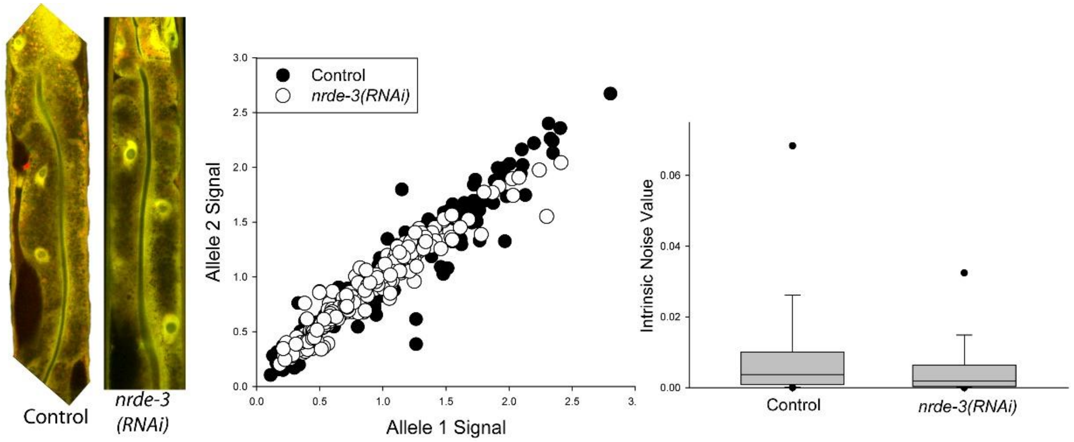

Supplemental Figure 2. Effect of nrde-3(RNAi) on MAE. Left: Images show $P_{hsp\text{-}90}$ reporter allele strain on EV or *nrde-3(RNAi).* Middle: Scatter plot of all cells. Right: Box plots of noise calculations show we detected no significant difference in intrinsic noise between EV and *nrde-3(RNAi)*, $P > 0.05$, Kruskal-Wallis One Way Analysis of Variance on Ranks followed by Dunn's Method, N=207 cells for control, N=209 cells form *nrde-3(RNAi)*, three independent experiments. Top of boxplot is 75th percentile, bottom of box is 25th percentile, line is median, top and bottom error bars are 90th and 10th percentile, respectively, and dots are 95th and 5th percentile.

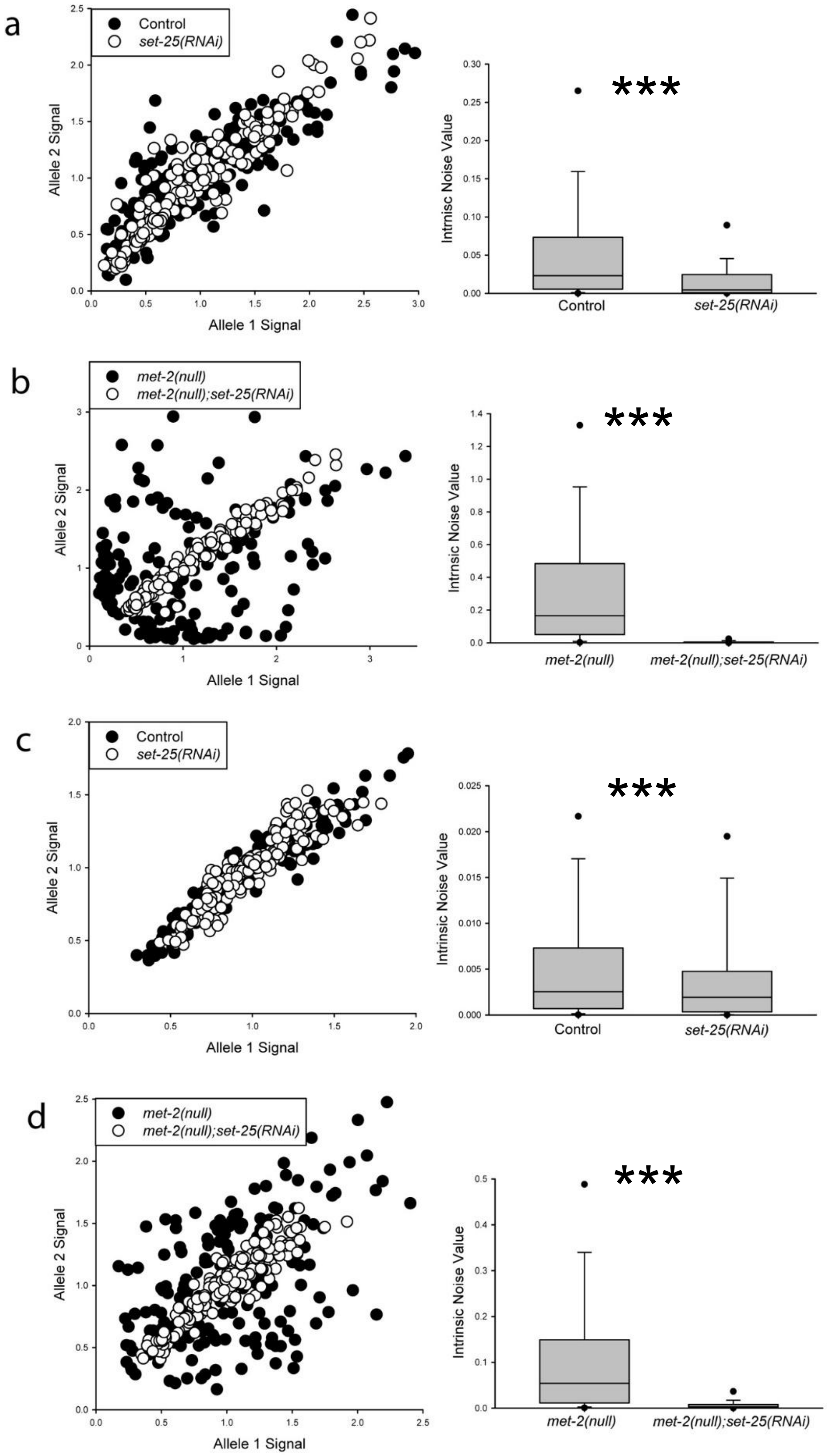

Supplemental Figure 3. Antagonistic control of aRMAE by met-2 and set-25 in eef1A and intronless reporter strains. a-d) Left panel shows scatter plot, right panel shows boxplot of intrinsic noise. Top of boxplot is 75th percentile, bottom of box is 25th percentile, line is median, top and bottom error bars are 90th and 10th percentile, respectively, and dots are 95th and 5th percentile. **a-b**) Intronless *hsp-90* reporter alleles. Intronless *hsp-90* reporter alleles have higher intrinsic noise than

hsp-90 alleles with introns, though the phenotype is still mostly BAE (See Sands et.al, 2021). **a**) Intronless *hsp-90* animals on *set-25* RNAi show decreased intrinsic noise, similar to main text Figure 4f. $P < 0.05$, Kruskal-Wallis One Way Analysis of Variance on Ranks followed by Dunnet's Test, N= 180 cells per group, three independent experiments. **b**) Intronless hsp-90 animals with *met-2(null)* mutation show an extreme aRMAE phenotype, which changes to extreme BAE on *set-25(RNAi)*, similar to our findings in main text Figure 4h. $P < 0.05$, Kruskal-Wallis One Way Analysis of Variance on Ranks followed by Dunnet's Test, N= 180 cells per group, three independent experiments. **c-d)**. *eef1A.1* reporter alleles show similar regulation of aRMAE by *met-2* and *set-25* as *hsp-90* reporter alleles (shown in main text Figure 4h). *eef1A.1* reporter alleles show an extreme BAE phenotype under normal conditions (see Sands et. Al, 2021). $P < 0.05$, Kruskal-Wallis One Way Analysis of Variance on Ranks followed by Dunnet's Test, N= 180 cells per group, three independent experiments. **c**) set-25 RNAi of *eef1A.1* reporter animals also results in extreme BAE. **d**) *eef1A.1* animals with *met-2(null)* show extreme aRMAE, which reverts back to BAE under *set-25* RNAi. $P < 0.05$, Kruskal-Wallis One Way Analysis of Variance on Ranks followed by Dunnet's Test, N= 180 cells per group, three independent experiments.

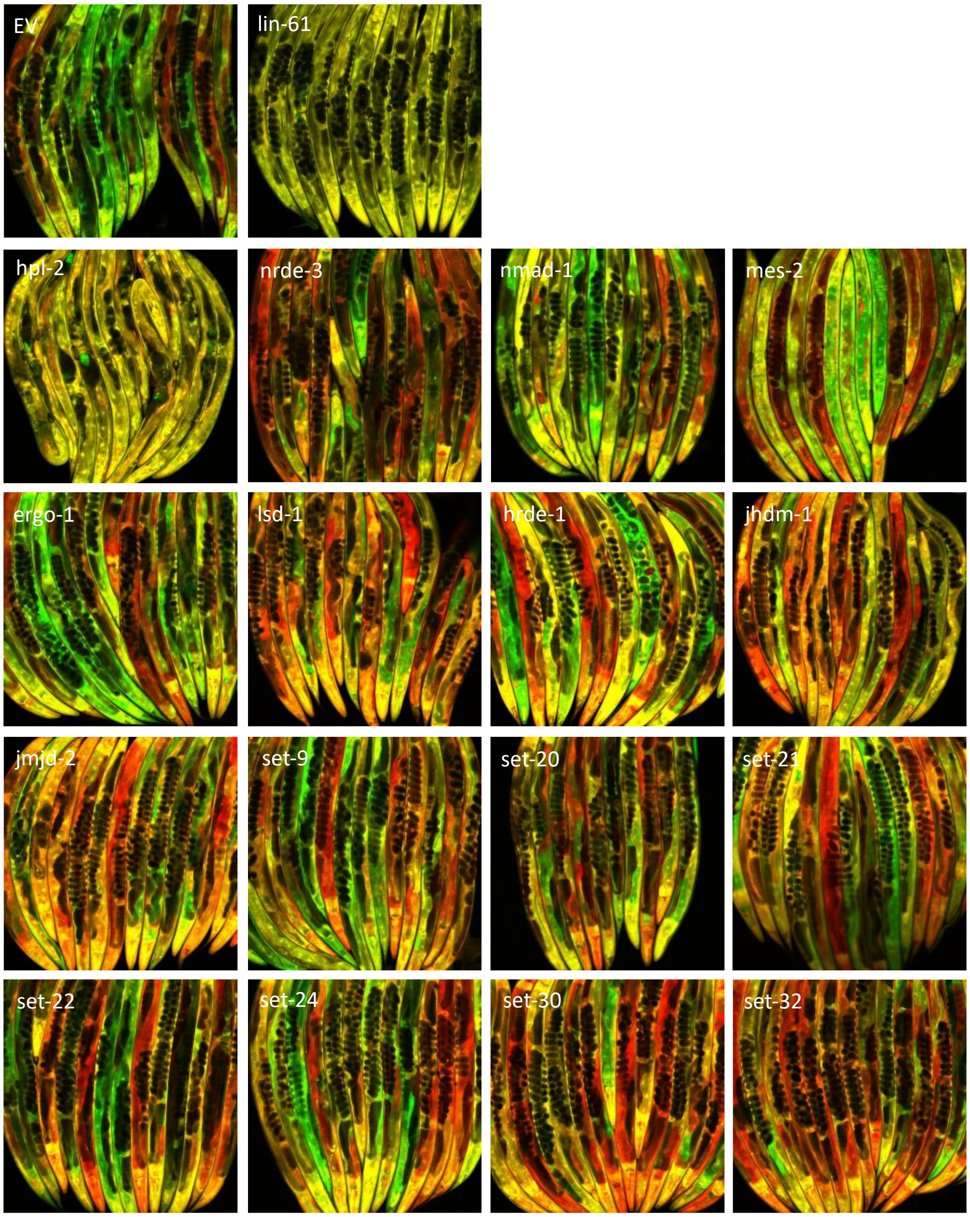

Supplemental Figure 4. **Suppressor screen in *met-2(null)* mutants**. Images are from RNAi screen of *met-2(null)* animals on indicated RNAi food. RNAi was conducted as described in Methods section. Images are from a Zeiss LSM 780 confocal microscope with a 10x air objective. Worms were anesthetized in round bottom 96 well plates, then transferred to a cover slip for imaging. Empty vector and *lin-61(RNAi)* were used as controls. Empty vector has no effect on aRMAE. *Lin-61* is a suppressor of aRMAE in the *met-2(null)* background, indicated by completely yellow worms expressing equal amounts of GFP and mCherry. The genes screened here all showed extreme BAE in our initial screen, similar to *set-25*.

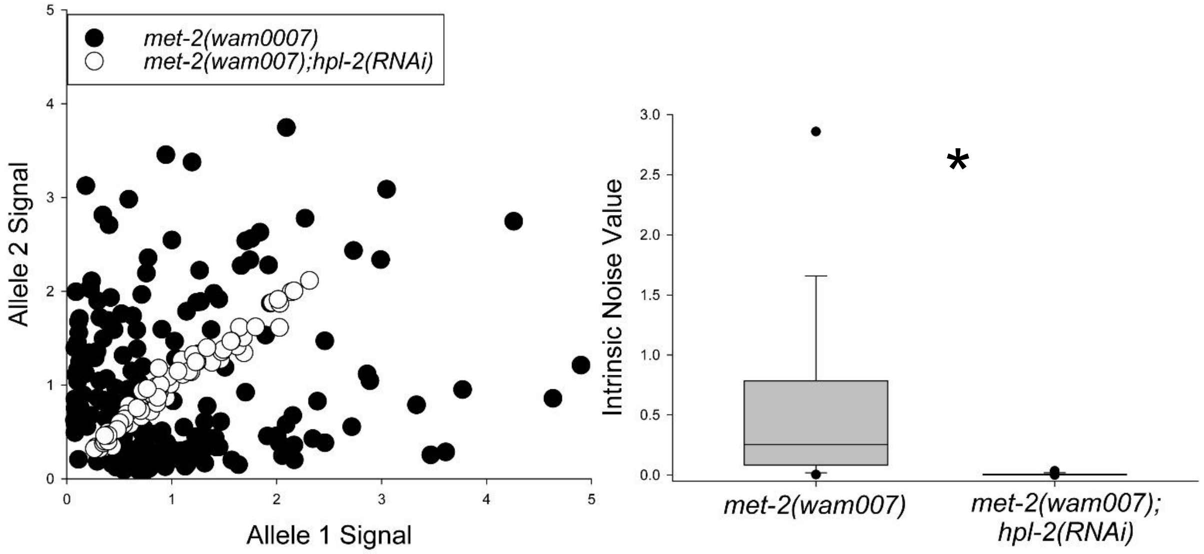

Supplemental Figure 5. Effects of *hpl-2* on *hsp-90* reporter allele expression in *met-2(null)* mutants. Left panel shows a scatter plot of *met-2(null)* animals' intestine cells plotted by allele expression level, compared to intestine cells in *met-2(null);hpl-2(RNAi)* animals. Right panel shows intrinsic noise quantified from the cells in the right panel during our suppressor screen. Top of boxplot is 75th percentile, bottom of box is 25th percentile, line is median, top and bottom error bars are 90th and 10th percentile, respectively, and dots are 95th and 5th percentile. There was a significant difference in intrinsic noise. $P < 0.05$, ANOVA on Ranks followed by Dunn's method for multiple comparison; N=70 cells for *hpl-2(RNAi)*, N=199 cells for *met-2(null)*. *met-2(null)* worms on *hpl-2(RNAi)* were imaged at 10x showing biallelic expression, similar to *set-25* and *lin-61* RNAi (see Supplemental Figure 3 and Figure 4). Here, we quantified expression from intestine as described in main text.

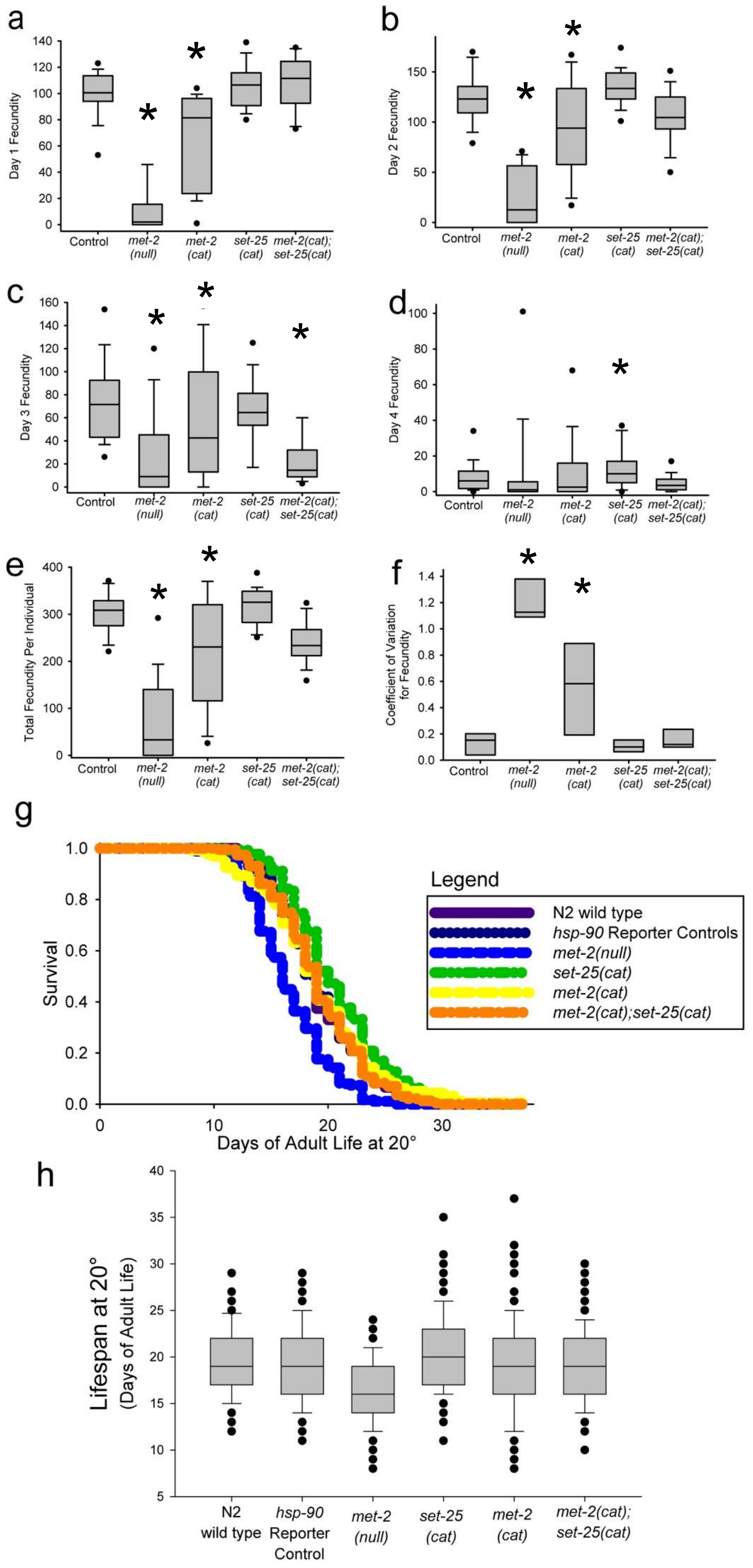

a
Day 1 Fecundity
Control
met-2 (null)
met-2 (cat)
set-25 (cat)
met-2(cat); set-25(cat)
b
Day 2 Fecundity
c
Day 3 Fecundity
d
Day 4 Fecundity
e
Total Fecundity Per Individual
f
Coefficient of Variation for Fecundity
g
Legend
N2 wild type
hsp-90 Reporter Controls
met-2(null)
set-25(cat)
met-2(cat)
met-2(cat);set-25(cat)
Survival
Days of Adult Life at 20°
h
Lifespan at 20° (Days of Adult Life)
N2 wild type
hsp-90 Reporter Control
met-2 (null)
set-25 (cat)
met-2 (cat)
met-2(cat); set-25(cat)

Supplemental Figure 6. MET-2 and SET-25 also regulates fecundity and aging. Boxplots of daily fecundity are shown in **a-d**. Boxplots show total fecundity for days 1-4 of adulthood in **e**, with interindividual variation in fecundity plotted in **f**. Top of boxplot is 75th percentile, bottom of box is 25th percentile, line is median, top and bottom error bars are 90th and 10th percentile, respectively, and dots are 95th and 5th percentile. **a)** shows progeny production on day 1 of adulthood. The *met-2* mutants had significantly lower progeny production than control animals. The *met-2(wam406);set-25(wam404)* animals had significantly higher progeny production than the *met-2(wam406)* animals on this day; $P < 0.05$ for noted comparisons, Tukey Test, N=18 per group, three independent experiments. **b)** shows progeny production on day 2 of adulthood. The *met-2* mutants still had significantly lower progeny production than wild type; $P < 0.05$ for both comparisons, Tukey Test, N=18 per group, three independent experiments. **c)** shows day 3 fecundity, with the same trend for *met-2* mutants persisting. The *met-2(wam406);set-25(wam404)* animals had significantly higher progeny production than the *met-2(wam406)* animals on this day; $P < 0.05$ for noted comparisons, Tukey Test, N=18 per group, three independent experiments. **d)** shows day 4 fecundity with almost no difference between groups. Interestingly, the *set-25* mutants had significantly more progeny than all other groups on day 4 of adulthood; $P < 0.05$, Student-Newman-Keuls Method, N=18 per group, three independent experiments. **e)** shows total fecundity for each group. Total fecundity was significantly lower than control animals for both *met-2* mutants; $P < 0.05$ for both comparisons, Student-Newman-Keuls Method, N=18 per group, three independent experiments. For total progeny, the double catalytic mutant, *met-2(wam406)*;s*et-25(wam404)* had significantly more progeny than the *met-2* knockout, *met-2(wam007)*, but not significantly more progeny than the *met-2* SET domain mutant, *met-2(wam406),* and significantly less than control and *set-25(wam404)* animals; $P < 0.05$, Student-Newman-Keuls Method, N=18 per group, three independent experiments. **f)** shows boxplots of the coefficient of variations from the individual experiments. There was significantly more interindividual variation in fecundity for the *met-2* mutants compared to control animals, as measured by the Coefficient of Variation (CV); $P < 0.05$ for both comparisons of CV, Holm-Sidak method, three experiments with three CV values per group. **g)** Lifespan line plots of different groups of animals are shown. X axis shows the days of adult life and the y axis shows the fraction of the population surviving. We did not detect a difference in median lifespan between N2 wild type or *hsp-90* reporter control animals median lifespan of 19 days and any other group besides *met-2(wam007)* animals, with a median lifespan of 16 days, and *set-25(wam404)* animals, with a median lifespan of 20 days; $P < 0.05$ for *met-2(null)* versus either control and *set-25(cat)* versus either control, Kruskal-Wallis One Way Analysis of Variance on Ranks followed by Dunns Method, N=182 for N2 wild type control, N=222 for *hsp-90* reporter control, N= 174 for *met-2(null)*, N=265 for *set-25(cat)*, N=160 for *met-2(cat)*, N=231 for *met-2(cat);set-25(cat),* six independent experiments. **h)** Data from lifespans in **g** as boxplots showing median lifespan as a line in the box, 25th and 75th percentile as vertical box bounds, 10th and 90th percentile as whisker bounds, and outliers as dots.

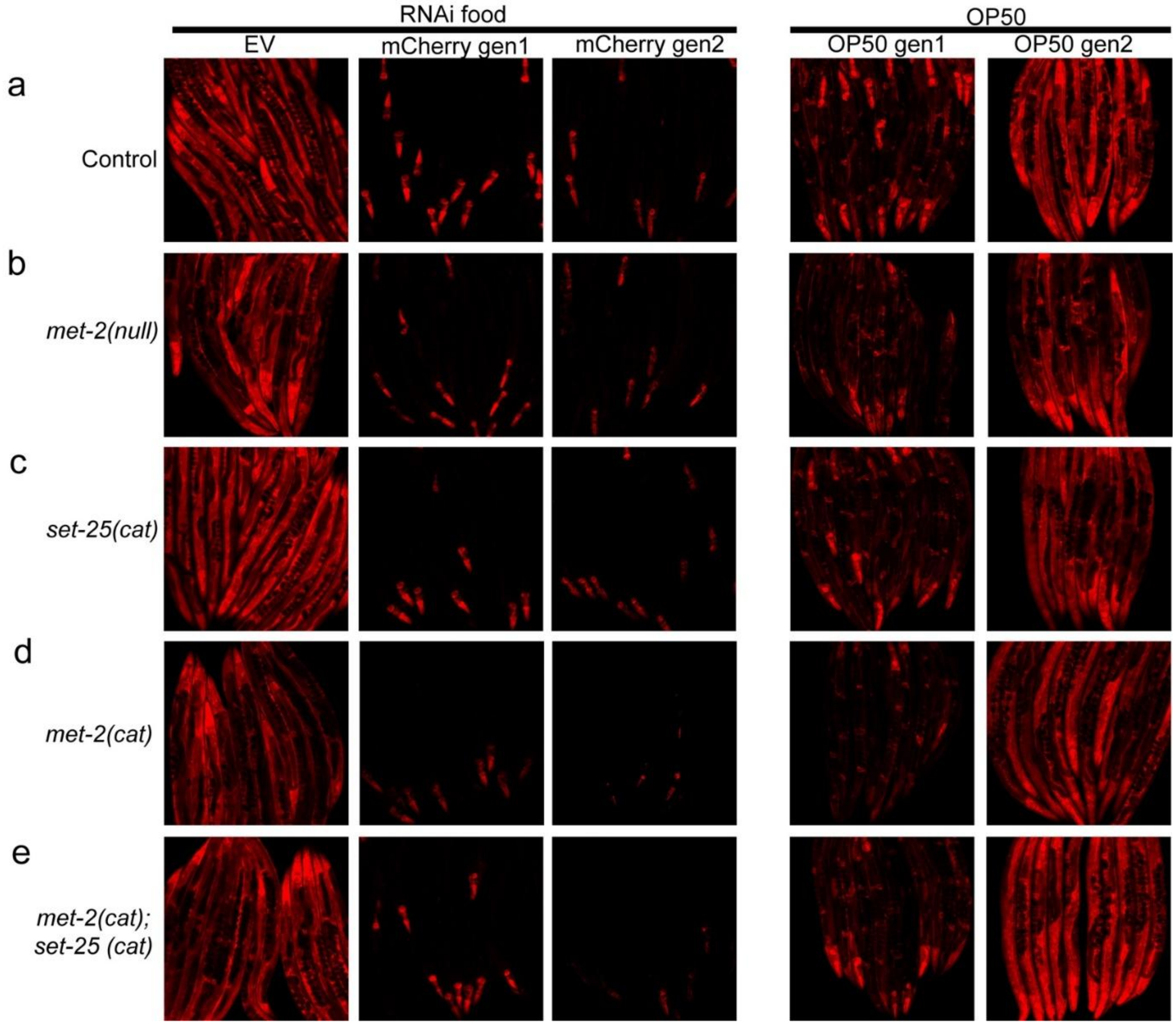

Supplemental Figure 7. **Somatic RNAi initiation and inheritance are not affected by MAE regulators**. Each row (**a-e**) shows a different genotype. Each column is a different generation. Images show the loss of mCherry signal over two generations of RNAi, and the reemergence of mCherry signal after two generations off of RNAi food. None of the conditions we tested showed any differences in the initiation or inheritance of RNAi. Images are representative of results from three independent experiments.

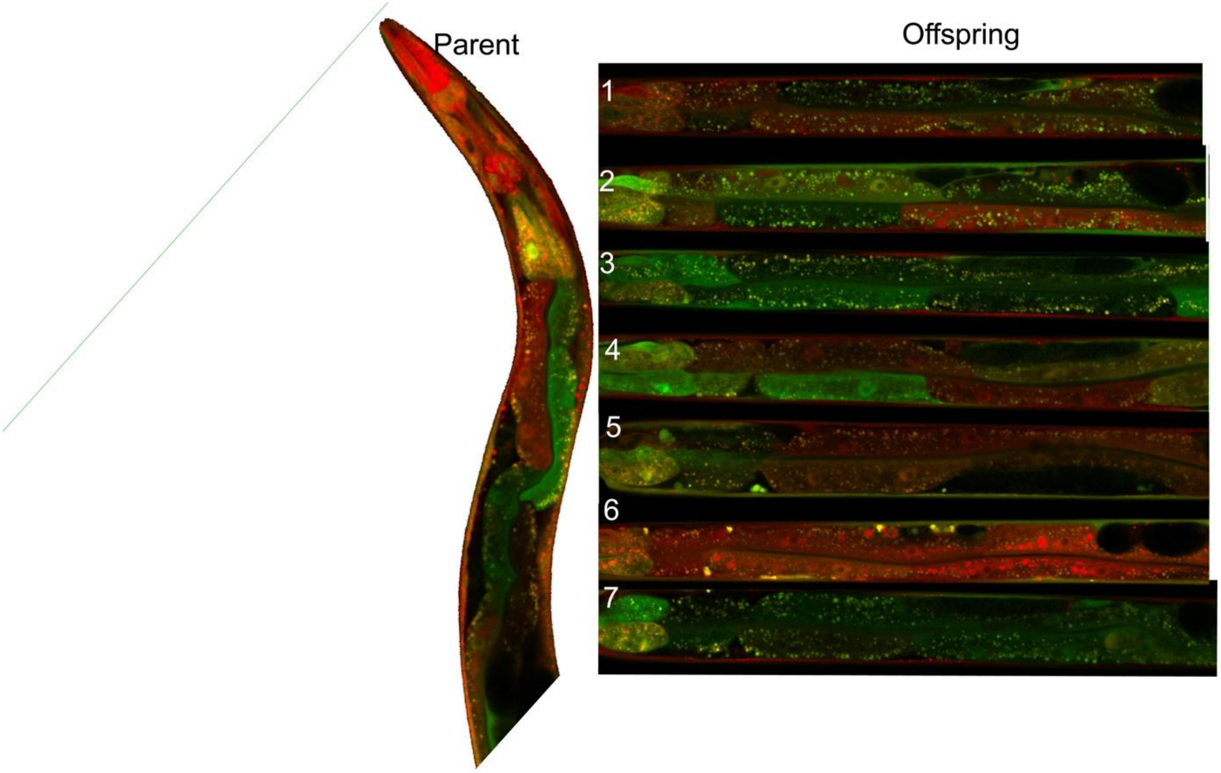

Supplemental Figure 8. **MAE is not heritable**. Image on left is a *met-2(wam406)* mutant parent hermaphrodite expressing differently colored *hsp-90* promoter controlled fluorescent alleles. Images on right are seven individual heterozygous progeny of the heterozygous parent. The heterozygous parent has a variegated pattern of expression and produces an array of diverse progeny with both variegated patterns and virtually monoallelic patterns of allele expression in the intestines. Results are representative of three independent experiments.

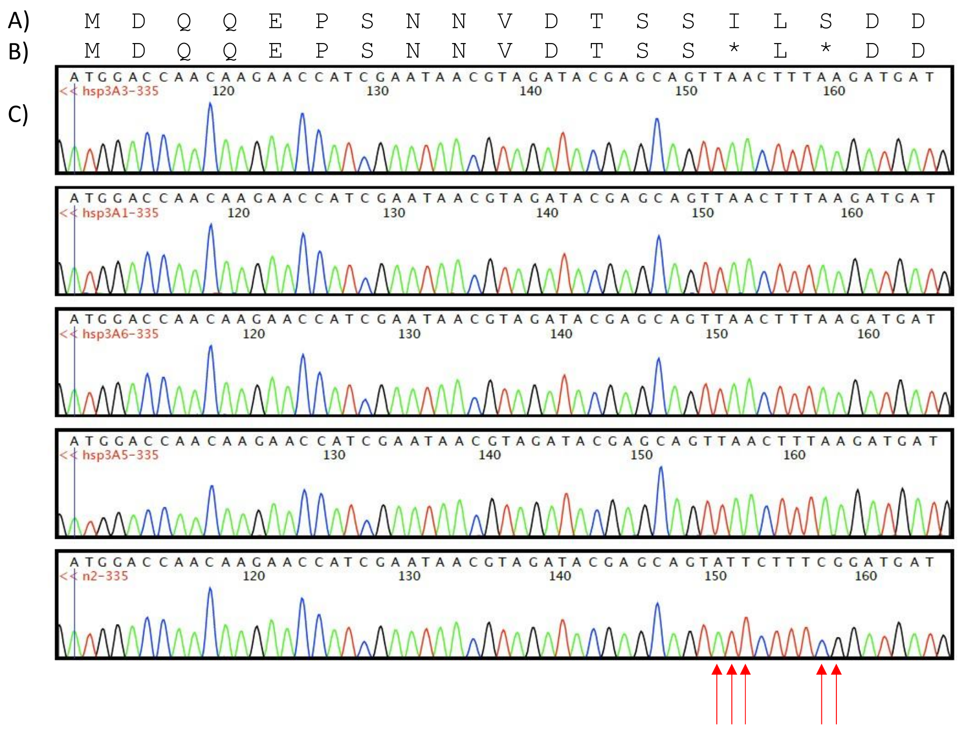

Supplemental Figure 9. Sequencing of CRISPR strains. Multiple independent CRISPR edits are sequenced. Pooled F3 animals are digested and a region that starts and ends outside the ssODN HDR region is amplified and Sanger sequenced in both direction (only top strand is shown here). Here, 4 independent hsp-90 CRISPR edits are sequenced along with an N2 control. All 4 show homozygous edits. There are 5 nt differences between the edited worms and N2 (arrows), creating 2 stop codons in frame with the ATG start codon of exon 1 in met-2. A) Protein sequence of wt MET-2 in N2 worms. B) Protein sequence of MET-2 after CRSIPR edit. C) AB1 trace file of 4 independent CRISPR edits and N2 as a control (lower panel). Note that there are no heterozygous edits or indels at the HDR site, indicating homozygous met-2 disruption.

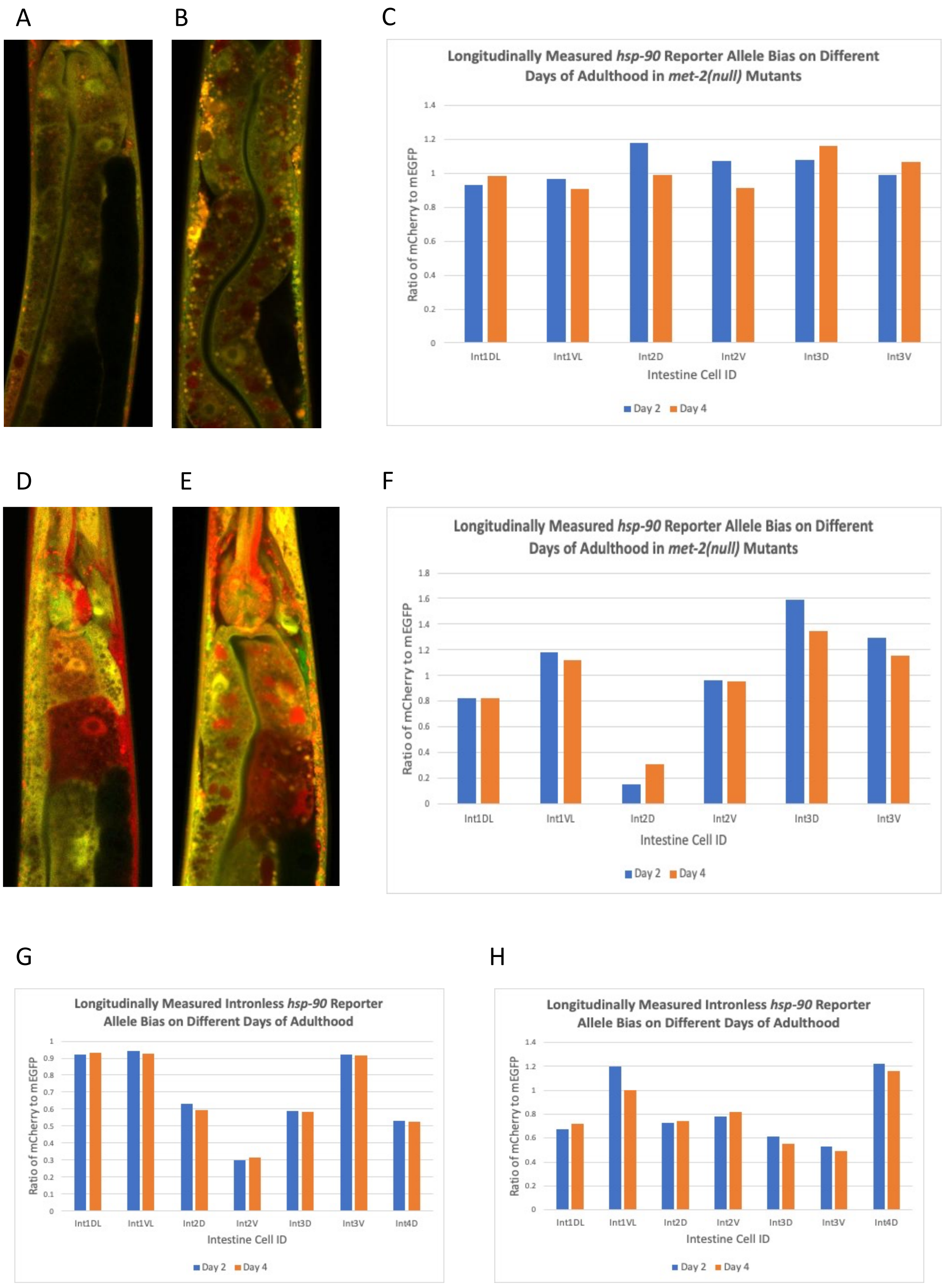

Supplemental Figure 10. Longitudinal analysis of aRMAE in *hsp-90* animals. Bar graphs show two individual ratiometric measurements performed on a single cell on two different days of adult life as blue (earlier) or orange (later) bars. **A-C**, *hsp-90* reporter alleles in a biallelic *met-2(KO)* worm. **A)** torso image of animal on D2 of adulthood. **B)** torso image of the same animal on D4. **C)** quantified gene expression of intestine cells. Ratio of GFP:mCherry does not change from D2 to D4 of adulthood. **D-F)**, *hsp-90* reporter alleles in a monoallelic *met-2(KO)* worm. **D)** torso image of animal on D2 of adulthood. **E)** torso image of the same animal on D4. **F)** quantified gene expression of intestine cells. Ratio of GFP:mCherry does not change from D2 to D4 of adulthood. Intestine cells that are monoallelic on D2 remain monoallelic on D4. **G-H)** quantified gene expression from intestine cells from *wt* worms with intronless *hsp-90* reporter alleles. In wt background, cells that are monoallelic on

D2 remain monoallelic on D4. Cells that are biallelic on D2 remain biallelic on D4. **G** and **H** each show a different individual. Intronless reporter alleles were used because they have a higher likelihood of going monoallelic, albeit the vast majority of cells are biallelic (see Sands et al., 2021).

Supplemental Table 1. Strains

Strain Name	Genotype	Reference
ARM133	*hutSi2661[unc-119(+), P_{hsp-90}:: megfp w/ 3 synthetic introns::T_{unc-54}, II:8420158]*	Sands et al., 2021
ARM135	*hutSi2642[unc-119(+), P_{hsp-90}::mcherry w/3 synthetic introns::T_{unc-54}, II:8420158]*	Sands et al., 2021
ARM148	*hutSi2581[unc-119(+), P_{vit-2}::mcherry w/ 3 synthetic introns::T_{unc-54}, II:8420158]*	Sands et al., 2021
ARM146	*hutSi2621[unc-119(+), P_{vit-2}::megfp w/ 3 synthetic introns::T_{unc-54}, II:8420158]*	Sands et al., 2021
ARM140	*hutSi2561[unc-119(+), $P_{hsp-16.2}$::mcherry w/ 3 synthetic introns::T_{unc-54}, II:8420158]*	Sands et al., 2021
ARM141	*hutSi2601[unc-119(+), $P_{hsp-16.2}$::megfp w/ 3 synthetic introns::T_{unc-54}, II:8420158]*	Sands et al., 2021
ARM284	*wamSi284[unc-119(+), P_{hsp-90}::mcherry w/3 synthetic introns::$T_{unc-54,}$ V:8643273]*	Sands et al., 2021
ARM291	*wamSi291[unc-119(+), P_{hsp-90}::megfp w/3 synthetic introns::$T_{unc-54,}$ V:8643273]*	Sands et al., 2021
ARM268	*wamSi268[unc-119(+), P_{hsp-90}::hsp-90 natural introns:: t2a :: mcherry ::$T_{unc-54,}$ II:8420158]*	Sands et al., 2021
ARM263	*wamSi263[unc-119(+), P_{hsp-90}::hsp-90 natural introns::t2a:: megfp ::$T_{unc-54,}$ II:8420158]*	Sands et al., 2021
ARM6	*wamSi6[unc-119(+), $P_{eef-1A.1}$::mtagBFP2 w/ 3 synthetic introns::T_{unc-54}, II:8420158]*	Sands et al., 2018
ARM3	*wamSi3[unc-119(+), $P_{eef-1A.1}$::mNeptune w/ 3 synthetic introns::T_{unc-54}, II:8420158]*	Sands et al., 2018
ARM366	*Idh-1(wam366[idh-1::T2A::mEGFP])*	This work
ARM379	*Idh-1(wam379[idh-1::T2A::mCherry])*	This work
ARM243 *met-2(Null)*	*met-2(wam007[I15stop]; [S17stop])*	This work
ARM406 *met-2(cat)*	*met-2(wam406[C1237A[)*	This work
ARM404	*set-25(wam404[C645A])*	This work

set-25(cat)		
ARM413 *met-2(cat); set-25(cat)*	*met-2(wam406[C1237A]; set-25(wam404[C645A])*	This work

Supplemental Table 2. CRISPR details

Edit:
met-2STOP
crRNA target:
AGATACGAGCAGTATTCTTTCGG
Repair Template:
ACAGCAGTGACGAATGAACTTTGTTCCTGTGTTTCCATCCCATCATCTTAAAGTTAACTGCTCGTATC
TACGTTATTCGATGGTTCTTGTTGGTCCATCT

Edit:
met-2cat
crRNA target:
ACGTGTTGAACGTGCACATTCGG
Repair Template:
GATACAATCATAAATTTTCGATAACTTTCAGATTCTTGAATCACTCTGCAGATCCGAACGTTCATGTGC
AGCATGTCATGTACGATACGCATGATCTTCGTCTTCCATGG

Edit:
Set-25cat
crRNA target:
ACTTCGACGAACACCGAGCTGGG
Repair Template:
GCAATAAAAAATTATTTCAGGAATATCTCCCGATTCATCAATCACAGCGCTGACCCTTCGAGTGTGTTC
GTCGAAGTCTACAGTCGACGATTCGAAGAAGATCCACTGATTCCAC

Edit:
Idh-1::GFP
crRNA target:
GGAATGAGAAAGTACTTAATGGG
Repair Template:
AAATTGGCTGAGAACCTCGCCAAGAAGCAAGCCCATGGATCTGGAGAGGGACGTGGATCCCTTCTTACCTGCGGAGACG
TCGAGGAGAACCCAGGACCAATGAGTAAAGGAGAAGAACTTTTCACTGGAGTTGTCCCAATTCTTGTTGAATTAGATGGT
GATGTTAATGGGCACAAATTTTCTGTCAGTGGAGAGGGTGAAGGTGATGCAACATACGGAAAACTTACCCTTAAATTTAT
TTGCACTACTGGAAAACTACCTGTTCCATGGCCAACACTTGTCACTACTTTCACTTATGGTGTTCAATGCTTTTCAAGATAC
CCAGATCATATGAAACGGCATGACTTTTTCAAGAGTGCCATGCCCGAAGGTTATGTACAGGAAAGAACTATATTTTTCAA
AGATGACGGGAACTACAAGACACGTGCTGAAGTCAAGTTTGAAGGTGATACCCTTGTTAATAGAATCGAGTTAAAAGGT
ATTGATTTTAAAGAAGATGGAAACATTCTTGGACACAAATTGGAATACAACTATAACTCACACAATGTATACATCATGGCA
GACAAACAAAAGAATGGAATCAAAGTTAACTTCAAAACTAGACACAACATTGAAGATGGAAGCGTTCAACTAGCAGACC
ATTATCAACAAAATACTCCAATTGGCGATGGCCCTGTCCTTTTACCAGACAACCATTACCTGTCCACACAATCTAAGCTTTC
GAAAGATCCCAACGAAAAGAGAGACCACATGGTCCTTCTTGAGTTTGTAACAGCTGCTGGGATTACACATGGCATGGAT
GAACTATACAAATAAGTACTTTCTCATTCCCAATCTAAAAATCTCGC

Edit:
Idh-1::cherry
crRNA target:
GGAATGAGAAAGTACTTAATGGG
Repair Template:
AAATTGGCTGAGAACCTCGCCAAGAAGCAAGCCCATGGATCTGGAGAGGGACGTGGATCCCTTCTTACCTGCGGAGACG
TCGAGGAGAACCCAGGACCAATGGTCTCAAAGGGTGAAGAAGATAACATGGCAATTATTAAAGAGTTTATGCGTTTCAA

GGTGCATATGGAGGGATCTGTCAATGGGCATGAGTTTGAAATTGAAGGTGAAGGAGAAGGCCGACCATATGAGGGAAC
ACAAACCGCAAAACTAAAGGTAACTAAAGGCGGACCATTACCATTCGCCTGGGACATCCTCTCTCCACAGTTCATGTATG
GAAGTAAAGCTTATGTTAAACATCCGGCAGATATACCAGATTATTTGAAACTTTCATTCCCGGAGGGTTTTAAGTGGGAA
CGCGTAATGAATTTTGAAGACGGAGGAGTTGTTACAGTGACGCAAGACTCAAGCCTCCAAGATGGAGAATTTATTTATAA
AGTCAAACTTCGAGGAACGAATTTCCCCTCGGATGGACCTGTTATGCAGAAGAAGACTATGGGATGGGAAGCTTCAAGT
GAAAGAATGTACCCTGAAGACGGTGCTCTTAAGGGAGAGATTAAACAACGTCTTAAATTGAAAGATGGAGGACATTACG
ATGCTGAGGTGAAGACAACTTACAAAGCCAAAAAACCAGTTCAGCTGCCAGGAGCGTACAATGTTAATATTAAACTGGAT
ATCACCTCCCACAACGAGGATTACACTATCGTTGAGCAATATGAAAGAGCTGAAGGGCGGCACTCGACAGGTGGCATGG
ATGAATTGTATAAGTAAGTACTTTCTCAT

Supplemental Table 3. Crosses

Crosses	Description of heterozygous animals
ARM135 x ARM133♂	*hsp-90* @ chr. II locus
ARM148 x ARM146♂	*vit-2* @ chr. II locus
ARM140 x ARM141♂	hsp-16.2 @ chr. II locus
ARM284 x ARM291♂	hsp-90 @ chr. V locus
ARM268 x ARM263♂	HSP90-T2A @ chr. II locus
ARM3 x ARM6♂	eef-1A.1 @ chr. II locus
ARM406♂ x N2	Paternal met-2 contribution
ARM406 x N2♂	Maternal met-2 contribution
ARM406♂ x ARM413	Maternal set-25 contribution
ARM406 x ARM413♂	Paternal set-25 contribution

Section 2 Statistical Analyses for All Figures

Figures 1 and 4 and Supplemental Figure 2

One Way Analysis of Variance

Normality Test (Shapiro-Wilk) Failed (P < 0.050)

Kruskal-Wallis One Way Analysis of Variance on Ranks

Group	N	Missing	Median	25%	75%
EV	207	0	0.00371	0.000859	0.0101
met-2	208	0	0.0607	0.00748	0.253
set-25	208	0	0.00109	0.000311	0.00299
lin-65	207	0	0.122	0.0257	0.497
arle-14	210	0	0.0110	0.00235	0.0250
lin-61	210	0	0.00105	0.000322	0.00289
cec-4	210	0	0.00467	0.00106	0.0123
lem-2	210	0	0.00624	0.00125	0.0209
nrde-3	209	0	0.00189	0.000335	0.00639

H = 611.498 with 8 degrees of freedom. (P = <0.001)

The differences in the median values among the treatment groups are greater than would be expected by chance; there is a statistically significant difference (P = <0.001)

To isolate the group or groups that differ from the others use a multiple comparison procedure.

All Pairwise Multiple Comparison Procedures (Dunn's Method) :

Comparison	Diff of Ranks	Q	P<0.05
lin-65 vs lin-61	934.564	17.587	Yes
lin-65 vs set-25	920.185	17.275	Yes
lin-65 vs nrde-3	815.497	15.328	Yes
lin-65 vs EV	665.715	12.483	Yes
lin-65 vs cec-4	607.440	11.431	Yes
lin-65 vs lem-2	530.421	9.982	Yes
lin-65 vs arle-14	436.736	8.219	Yes
lin-65 vs met-2	127.848	2.400	No
met-2 vs lin-61	806.716	15.199	Yes
met-2 vs set-25	792.337	14.893	Yes
met-2 vs nrde-3	687.649	12.941	Yes
met-2 vs EV	537.866	10.098	Yes
met-2 vs cec-4	479.592	9.036	Yes
met-2 vs lem-2	402.573	7.585	Yes
met-2 vs arle-14	308.887	5.820	Yes
arle-14 vs lin-61	497.829	9.402	Yes
arle-14 vs set-25	483.449	9.109	Yes
arle-14 vs nrde-3	378.762	7.145	Yes
arle-14 vs EV	228.979	4.309	Yes
arle-14 vs cec-4	170.705	3.224	Yes
arle-14 vs lem-2	93.686	1.769	No
lem-2 vs lin-61	404.143	7.633	Yes
lem-2 vs set-25	389.764	7.343	Yes
lem-2 vs nrde-3	285.076	5.378	Yes
lem-2 vs EV	135.294	2.546	No
lem-2 vs cec-4	77.019	1.455	Do Not Test
cec-4 vs lin-61	327.124	6.178	Yes
cec-4 vs set-25	312.745	5.892	Yes
cec-4 vs nrde-3	208.057	3.925	Yes
cec-4 vs EV	58.275	1.097	Do Not Test

EV vs lin-61	268.849	5.059	Yes
EV vs set-25	254.470	4.777	Yes
EV vs nrde-3	.714983	2.815	No
nrde-3 vs lin-61	119.067	2.246	No
nrde-3 vs set-25	104.688	1.970	Do Not Test
set-25 vs lin-61	14.379	0.271	Do Not Test

Note: The multiple comparisons on ranks do not include an adjustment for ties.

Figures 4g&h and Supplemental Figure 4

Normality Test (Shapiro-Wilk) Failed (P < 0.050)

Group	N	Missing	Median	25%	75%
met-2ko	199	0	0.254	0.0840	0.784
met-2ko;set-25RNAi	204	0	0.00237	0.000525	0.0819
met2 lin61	210	0	0.00160	0.000328	0.00412
met-2; hpl-2rnai	70	0	0.00363	0.000912	0.00854

H = 285.132 with 3 degrees of freedom. (P = <0.001)

The differences in the median values among the treatment groups are greater than would be expected by chance; there is a statistically significant difference (P = <0.001)

To isolate the group or groups that differ from the others use a multiple comparison procedure.

All Pairwise Multiple Comparison Procedures (Dunn's Method) :

Comparison	Diff of Ranks	Q	P<0.05
met-2ko vs met2 lin61	314.943	16.135	Yes
met-2ko vs met-2; hpl-2rnai	258.924	9.443	Yes
met-2ko vs met-2ko;set-25RNAi	228.846	11.641	Yes
met-2ko;set-2 vs met2 lin61	86.096	4.439	Yes
met-2ko;set-2 vs met-2; hpl-2r	30.077	1.100	No
met-2; hpl-2rnai vs met2 lin61	56.019	2.057	No

Note: The multiple comparisons on ranks do not include an adjustment for ties.

Figure 2a

Mann-Whitney Rank Sum Test

Normality Test (Shapiro-Wilk) Failed (P < 0.050)

Group	N	Missing	Median	25%	75%
Control	209	0	0.00232	0.000737	0.00770
met-2 ko	208	0	0.187	0.0318	0.465

Mann-Whitney U Statistic= 4353.000

T = 60855.000 n(small)= 208 n(big)= 209 (P = <0.001)

The difference in the median values between the two groups is greater than would be expected by chance; there is a statistically significant difference (P = <0.001)

Figure 2c

Mann-Whitney Rank Sum Test

Normality Test (Shapiro-Wilk) Failed (P < 0.050)

Group	N	Missing	Median	25%	75%
control t2a	196	0	0.00163	0.000292	0.00611
met-2 ko t2a	210	0	0.100	0.0166	0.352

Mann-Whitney U Statistic= 4215.000

T = 23521.000 n(small)= 196 n(big)= 210 (P = <0.001)

The difference in the median values between the two groups is greater than would be expected by chance; there is a statistically significant difference (P = <0.001)

Figure 2b

Mann-Whitney Rank Sum Test
Normality Test (Shapiro-Wilk) Failed (P < 0.050)

Group	N	Missing	Median	25%	75%
EV chr V	207	0	0.0196	0.00363	0.0519
CHR V met-2 RNAi	206	0	0.180	0.0552	0.551

Mann-Whitney U Statistic= 8475.000

T = 55488.000 n(small)= 206 n(big)= 207 (P = <0.001)

The difference in the median values between the two groups is greater than would be expected by chance; there is a statistically significant difference (P = <0.001)

Figure 3a

Mann-Whitney Rank Sum Test
Normality Test (Shapiro-Wilk) Failed (P < 0.050)

Group	N	Missing	Median	25%	75%
vit-2	210	0	0.000968	0.000319	0.00436
met-2 ko vit2	209	0	0.000809	0.000246	0.00275

Mann-Whitney U Statistic= 20567.000

T = 42512.000 n(small)= 209 n(big)= 210 (P = 0.266)

The difference in the median values between the two groups is not great enough to exclude the possibility that the difference is due to random sampling variability; there is not a statistically significant difference (P = 0.266)

Figure 3b

Mann-Whitney Rank Sum Test

Normality Test (Shapiro-Wilk) Failed (P < 0.050)

Group	N	Missing	Median	25%	75%
16.2 ev	210	0	0.00297	0.000699	0.00783
16.2 met2 RNAi	210	0	0.00311	0.000754	0.00994

Mann-Whitney U Statistic= 21389.000

T = 43544.000 n(small)= 210 n(big)= 210 (P = 0.595)

The difference in the median values between the two groups is not great enough to exclude the possibility that the difference is due to random sampling variability; there is not a statistically significant difference (P = 0.595)

Figure 3c

Mann-Whitney Rank Sum Test

Normality Test (Shapiro-Wilk) Failed (P < 0.050)

Group	N	Missing	Median	25%	75%
idh-1 ev	208	0	0.00356	0.00104	0.0123
idh-1 met-2 RNAi	209	0	0.00337	0.000888	0.0151

Mann-Whitney U Statistic= 21311.000

T = 43897.000 n(small)= 208 n(big)= 209 (P = 0.730)

The difference in the median values between the two groups is not great enough to exclude the possibility that the difference is due to random sampling variability; there is not a statistically significant difference (P = 0.730)

Figure 3d

Mann-Whitney Rank Sum Test

Normality Test (Shapiro-Wilk) Failed (P < 0.050)

Group	N	Missing	Median	25%	75%
EV EFT-3	180	0	0.00254	0.000697	0.00730
eft-3 met-2 RNAi	180	0	0.00944	0.00248	0.0339

Mann-Whitney U Statistic= 9359.000

T = 25649.000 n(small)= 180 n(big)= 180 (P = <0.001)

The difference in the median values between the two groups is greater than would be expected by chance; there is a statistically significant difference (P = <0.001)

Figure 5

Kruskal-Wallis One Way Analysis of Variance on Ranks

Normality Test (Shapiro-Wilk) Failed (P < 0.050)

Group	N	Missing	Median	25%	75%
control	209	0	0.00459	0.000925	0.0144
404	210	0	0.00114	0.000288	0.00381
406	210	0	0.134	0.0237	0.469
414	210	0	0.00140	0.000351	0.00438

H = 355.298 with 3 degrees of freedom. (P = <0.001)

The differences in the median values among the treatment groups are greater than would be expected by chance; there is a statistically significant difference (P = <0.001)

To isolate the group or groups that differ from the others use a multiple comparison procedure.

All Pairwise Multiple Comparison Procedures (Dunn's Method) :

Comparison	Diff of Ranks	Q	P<0.05
406 vs 404	396.676	16.773	Yes
406 vs 414	374.019	15.815	Yes
406 vs control	269.196	11.369	Yes
control vs 404	127.480	5.384	Yes
control vs 414	104.823	4.427	Yes
414 vs 404	22.657	0.958	No

Note: The multiple comparisons on ranks do not include an adjustment for ties.

Supplemental Figure 6a

Day 1 Adults

Kruskal-Wallis One Way Analysis of Variance on Ranks

Normality Test (Shapiro-Wilk) Passed (P = 0.572)

Equal Variance Test: Failed (P < 0.050)

Group	N	Missing	Median	25%	75%
hsp90	18	0	100.500	94.000	113.500
met-2KO	18	0	2.000	0.000	15.500
met-2cat	18	0	81.500	23.750	96.250
set-25cat	18	0	106.500	90.750	115.750
doublecat	18	0	111.500	92.500	124.500

H = 53.867 with 4 degrees of freedom. (P = <0.001)

The differences in the median values among the treatment groups are greater than would be expected by chance; there is a statistically significant difference (P = <0.001)

To isolate the group or groups that differ from the others use a multiple comparison procedure.

All Pairwise Multiple Comparison Procedures (Tukey Test):

Comparison	Diff of Ranks	q	P<0.05
doublecat vs met-2KO	946.000	8.535	Yes
doublecat vs met-2cat	530.000	4.782	Yes
doublecat vs hsp90	132.000	1.191	No
doublecat vs set-25cat	24.500	0.221	Do Not Test
set-25cat vs met-2KO	921.500	8.314	Yes
set-25cat vs met-2cat	505.500	4.561	Yes
set-25cat vs hsp90	107.500	0.970	Do Not Test
hsp90 vs met-2KO	814.000	7.344	Yes
hsp90 vs met-2cat	398.000	3.591	No
met-2cat vs met-2KO	416.000	3.753	No

Note: The multiple comparisons on ranks do not include an adjustment for ties.

A result of "Do Not Test" occurs for a comparison when no significant difference is found between the two rank sums that enclose that comparison. For example, if you had four rank sums sorted in order, and found no significant difference between rank sums 4 vs. 2, then you would not test 4 vs. 3 and 3 vs. 2, but still test 4 vs. 1 and 3 vs. 1 (4 vs. 3 and 3 vs. 2 are enclosed by 4 vs. 2: 4 3 2 1). Note that not testing the enclosed rank sums is a procedural rule, and a result of Do Not Test should be treated as if there is no significant difference between the rank sums, even though one may appear to exist.

Supplemental Figure 6b

Day 2 Adults

Kruskal-Wallis One Way Analysis of Variance on Ranks

Normality Test (Shapiro-Wilk) Passed (P = 0.684)

Equal Variance Test: Failed (P < 0.050)

Group	N	Missing	Median	25%	75%
hsp90	18	0	123.000	109.250	135.500
met-2KO	18	0	12.500	0.000	56.500
met-2cat	18	0	94.000	57.750	133.500
set-25cat	18	0	133.500	123.000	149.000
double cat	18	0	104.500	93.250	125.000

H = 48.032 with 4 degrees of freedom. (P = <0.001)

The differences in the median values among the treatment groups are greater than would be expected by chance; there is a statistically significant difference (P = <0.001)

To isolate the group or groups that differ from the others use a multiple comparison procedure.

All Pairwise Multiple Comparison Procedures (Tukey Test):

Comparison	Diff of Ranks	q	P<0.05
set-25cat vs met-2KO	1023.000	9.230	Yes
set-25cat vs met-2cat	448.500	4.046	Yes
set-25cat vs double cat	417.000	3.762	No
set-25cat vs hsp90	199.000	1.795	Do Not Test
hsp90 vs met-2KO	824.000	7.434	Yes
hsp90 vs met-2cat	249.500	2.251	No
hsp90 vs double cat	218.000	1.967	Do Not Test
double cat vs met-2KO	606.000	5.467	Yes
double cat vs met-2cat	31.500	0.284	Do Not Test
met-2cat vs met-2KO	574.500	5.183	Yes

Note: The multiple comparisons on ranks do not include an adjustment for ties.

A result of "Do Not Test" occurs for a comparison when no significant difference is found between the two rank sums that enclose that comparison. For example, if you had four rank sums sorted in order, and found no significant difference between rank sums 4 vs. 2, then you would not test 4 vs. 3 and 3 vs. 2, but still test 4 vs. 1 and 3 vs. 1 (4 vs. 3 and 3 vs. 2 are enclosed by 4 vs. 2: 4 3 2 1). Note that not testing the enclosed rank sums is a procedural rule, and a result of Do Not Test should be treated as if there is no significant difference between the rank sums, even though one may appear to exist.

Supplemental Figure 6c

Day 3 Adults

Kruskal-Wallis One Way Analysis of Variance on Ranks

Normality Test (Shapiro-Wilk) Failed (P < 0.050)

Group	N	Missing	Median	25%	75%
hsp90	18	0	71.500	43.000	92.500
met-2KO	18	0	9.000	0.000	45.250
met-2cat	18	0	42.500	13.000	99.750
set-25cat	18	0	64.500	53.500	81.250

double cat	18	0	14.500	8.750	32.000

H = 31.125 with 4 degrees of freedom. (P = <0.001)

The differences in the median values among the treatment groups are greater than would be expected by chance; there is a statistically significant difference (P = <0.001)

To isolate the group or groups that differ from the others use a multiple comparison procedure.

All Pairwise Multiple Comparison Procedures (Student-Newman-Keuls Method) :

**Comparison**	**Diff of Ranks**	**q**	**P<0.05**
hsp90 vs met-2KO	639.000	5.765	Yes
hsp90 vs double cat	636.500	7.168	Yes
hsp90 vs met-2cat	283.000	4.240	Yes
hsp90 vs set-25cat	44.000	0.984	No
set-25cat vs met-2KO	595.000	6.701	Yes
set-25cat vs double cat	592.500	8.877	Yes
set-25cat vs met-2cat	239.000	5.347	Yes
met-2cat vs met-2KO	356.000	5.334	Yes
met-2cat vs double cat	353.500	7.908	Yes
double cat vs met-2KO	2.500	0.0559	No

Note: The multiple comparisons on ranks do not include an adjustment for ties.

Supplemental Figure 6d

Day 4 Adults

Kruskal-Wallis One Way Analysis of Variance on Ranks

Normality Test (Shapiro-Wilk) Failed (P < 0.050)

**Group**	**N**	**Missing**	**Median**	**25%**	**75%**
hsp90	18	0	6.000	1.750	11.500
met-2KO	18	0	1.000	0.000	5.500
met-2cat	18	0	2.500	0.000	16.000
set-25cat	18	0	10.000	5.000	17.000
doublecat	18	0	3.500	1.000	7.000

H = 12.823 with 4 degrees of freedom. (P = 0.012)

The differences in the median values among the treatment groups are greater than would be expected by chance; there is a statistically significant difference (P = 0.012)

To isolate the group or groups that differ from the others use a multiple comparison procedure.

All Pairwise Multiple Comparison Procedures (Student-Newman-Keuls Method) :

**Comparison**	**Diff of Ranks**	**q**	**P<0.05**
set-25cat vs met-2KO	509.000	4.592	Yes
set-25cat vs doublecat	380.500	4.285	Yes
set-25cat vs met-2cat	355.000	5.319	Yes
set-25cat vs hsp90	185.500	4.150	Yes
hsp90 vs met-2KO	323.500	3.643	Yes
hsp90 vs doublecat	195.000	2.922	No
hsp90 vs met-2cat	169.500	3.792	Do Not Test
met-2cat vs met-2KO	154.000	2.307	No
met-2cat vs doublecat	25.500	0.570	Do Not Test
doublecat vs met-2KO	128.500	2.875	Do Not Test

Note: The multiple comparisons on ranks do not include an adjustment for ties.

A result of "Do Not Test" occurs for a comparison when no significant difference is found between the two rank sums that enclose that comparison. For example, if you had four rank sums sorted in order, and found no significant difference between rank sums 4 vs. 2, then you would not test 4 vs. 3 and 3 vs. 2, but still test 4 vs. 1 and 3 vs. 1 (4 vs. 3 and 3 vs. 2 are enclosed by 4 vs. 2: 4 3 2 1). Note that not testing the enclosed rank sums is a procedural rule, and a result of Do Not Test should be treated as if there is no significant difference between the rank sums, even though one may appear to exist.

Supplemental Figure 6e

Total Progeny Per Animal

One Way Analysis of Variance
Normality Test (Shapiro-Wilk) Passed (P = 0.073)
Equal Variance Test: Failed (P < 0.050)
Kruskal-Wallis One Way Analysis of Variance on Ranks

Dependent Variable:

Group	N	Missing	Median	25%	75%
control	18	0	308.500	275.750	329.000
met-2 ko	18	0	33.000	0.000	140.000
met-2 cat	18	0	230.500	116.000	320.500
set-25 cat	18	0	325.500	282.750	349.000
met-2 cat;set-25 cat	18	0	233.500	212.000	267.500

H = 48.790 with 4 degrees of freedom. (P = <0.001)

The differences in the median values among the treatment groups are greater than would be expected by chance; there is a statistically significant difference (P = <0.001)

To isolate the group or groups that differ from the others use a multiple comparison procedure.

All Pairwise Multiple Comparison Procedures (Student-Newman-Keuls Method) :

Comparison	Diff of Ranks	q	P<0.05
set-25 cat vs met-2 ko	989.500	8.927	Yes
set-25 cat vs met-2 cat;set	519.000	5.845	Yes
set-25 cat vs met-2 cat	441.000	6.607	Yes
set-25 cat vs control	123.000	2.752	No
control vs met-2 ko	866.500	9.759	Yes
control vs met-2 cat;set	396.000	5.933	Yes
control vs met-2 cat	318.000	7.114	Yes
met-2 cat vs met-2 ko	548.500	8.218	Yes
met-2 cat vs met-2 cat;set	78.000	1.745	No
met-2 cat;set vs met-2 ko	470.500	10.526	Yes

Note: The multiple comparisons on ranks do not include an adjustment for ties.

Supplemental Figure 6f

Coefficient of Variation for Total Progeny Per Individual

Kruskal-Wallis One Way Analysis of Variance on Ranks

One Way Analysis of Variance

Group Name	N	Missing	Mean	Std Dev	SEM
control	3	0	0.131	0.0825	0.0477

met-2 ko	3	0	1.199	0.157	0.0908
met-2 cat	3	0	0.554	0.349	0.202
set-25 cat	3	0	0.106	0.0450	0.0260
met-2 cat;set-25 cat	3	0	0.151	0.0728	0.0420

**Source of Variation**	**DF**	**SS**	**MS**	**F**	**P**
Between Groups	4	2.633	0.658	20.462	<0.001
Residual	10	0.322	0.0322		
Total	14	2.955			

The differences in the mean values among the treatment groups are greater than would be expected by chance; there is a statistically significant difference (P = <0.001).

Power of performed test with alpha = 0.050: 1.000

Multiple Comparisons versus Control Group (Holm-Sidak method):
Overall significance level = 0.05

Comparisons for factor:

**Comparison**	**Diff of Means**	**t**	**P**	**P<0.050**
control vs. met-2 ko	1.067	7.288	<0.001	Yes
control vs. met-2 cat	0.423	2.889	0.048	Yes
control vs. set-25 cat	0.0248	0.169	0.983	No
control vs. met-2 cat;se	0.0203	0.139	0.892	No

Supplemental Figure 6g
Lifespan
Normality Test (Shapiro-Wilk) Failed (P < 0.050)

Kruskal-Wallis One Way Analysis of Variance on Ranks

**Group**	**N**	**Missing**	**Median**	**25%**	**75%**
N2	182	0	19.000	17.000	22.000
hsp90 ctrl	222	0	19.000	16.000	22.000
met-2(wam007)	174	0	16.000	14.000	19.000
set-25(wam404)	265	0	20.000	17.000	23.000
met-2(wam406)	160	0	19.000	16.000	22.000
double cat	231	0	19.000	16.000	22.000

H = 104.717 with 5 degrees of freedom. (P = <0.001)

The differences in the median values among the treatment groups are greater than would be expected by chance; there is a statistically significant difference (P = <0.001)

To isolate the group or groups that differ from the others use a multiple comparison procedure.

All Pairwise Multiple Comparison Procedures (Dunn's Method) :

**Comparison**	**Diff of Ranks**	**Q**	**P<0.05**
set-25(wam404 vs met-2(wam007)	352.841	10.147	Yes
set-25(wam404 vs met-2(wam406)	128.733	3.608	Yes
set-25(wam404) vs hsp90 ctrl	119.050	3.672	Yes
set-25(wam404) vs double cat	118.490	3.694	Yes
set-25(wam404) vs N2	113.763	3.316	Yes
N2 vs met-2(wam007)	239.078	6.327	Yes
N2 vs met-2(wam406)	14.970	0.388	No
N2 vs hsp90 ctrl	5.287	0.148	Do Not Test
N2 vs double cat	4.727	0.134	Do Not Test
double cat vs met-2(wam007)	234.351	6.551	Yes

double cat vs met-2(wam406)	10.244	0.279	Do Not Test
double cat vs hsp90 ctrl	0.561	0.0167	Do Not Test
hsp90 ctrl vs met-2(wam007)	233.790	6.479	Yes
hsp90 ctrl vs met-2(wam406)	9.683	0.262	Do Not Test
met-2(wam406) vs met-2(wam007)	224.108	5.741	Yes

Note: The multiple comparisons on ranks do not include an adjustment for ties.

Figure 6f

Mann-Whitney Rank Sum Test

Normality Test (Shapiro-Wilk) Failed (P < 0.050)

**Group**	**N**	**Missing**	**Median**	**25%**	**75%**
m2cat f	180	0	0.0469	0.00781	0.159
m2cat m	180	0	0.00193	0.000572	0.00737

Mann-Whitney U Statistic= 5438.000

T = 43252.000 n(small)= 180 n(big)= 180 (P = <0.001)

The difference in the median values between the two groups is greater than would be expected by chance; there is a statistically significant difference (P = <0.001)

Figure 6l

Mann-Whitney Rank Sum Test

Normality Test (Shapiro-Wilk) Failed (P < 0.050)

**Group**	**N**	**Missing**	**Median**	**25%**	**75%**
dc f	180	0	0.00109	0.000308	0.00267
dc m	180	0	0.0508	0.0122	0.154

Mann-Whitney U Statistic= 2279.000

T = 18569.000 n(small)= 180 n(big)= 180 (P = <0.001)

The difference in the median values between the two groups is greater than would be expected by chance; there is a statistically significant difference (P = <0.001)
